# Immunohistochemistry cannot replace DNA analysis for evaluation of *BRAF* V600E mutations in papillary thyroid carcinoma

**DOI:** 10.18632/oncotarget.20451

**Published:** 2017-08-24

**Authors:** Monika Szymonek, Artur Kowalik, Janusz Kopczyński, Danuta Gąsior-Perczak, Iwona Pałyga, Agnieszka Walczyk, Klaudia Gadawska-Juszczyk, Agnieszka Płusa, Ryszard Mężyk, Magdalena Chrapek, Stanisław Góźdź, Aldona Kowalska

**Affiliations:** ^1^ Endocrinology Clinic, Holycross Cancer Center, Kielce, Poland; ^2^ Department of Molecular Diagnostics, Holycross Cancer Center, Kielce, Poland; ^3^ Department of Surgical Pathology, Holycross Cancer Center, Kielce, Poland; ^4^ Cancer Epidemiology, Holycross Cancer Center, Kielce, Poland; ^5^ Department of Probability Theory and Statistics Institute of Mathematics, Faculty of Mathematics and Natural Science, Jan Kochanowski University, Kielce, Poland; ^6^ Oncology Clinic, Holycross Cancer Center, Kielce, Poland; ^7^ The Faculty of Health Sciences, Jan Kochanowski University in Kielce, Poland

**Keywords:** BRAF V600E, papillary thyroid cancer, immunohistochemistry, Sanger sequencing, qPCR

## Abstract

**Introduction:**

The *BRAF* V600E mutation is the most common genetic event occurring in papillary thyroid cancer (PTC). Recently, the possibility of using immunohistochemistry (IHC) to detect the *BRAF* V600E mutation has been reported.

**Materials and Methods:**

In 140 patients with classical PTC, the status of the *BRAF* V600E mutation was determined by IHC (using two alternative staining protocols, IHC-1 and IHC-2) and molecular biology methods: Sanger sequencing (SEQ) and real-time PCR (qPCR).

**Results:**

The *BRAF* V600E mutation was detected in 57.1% (80/140) patients by IHC-1 and 62.9% (88/140) patients by IHC-2. The highest correlation in detecting the *BRAF* V600E mutation was found between IHC-2 and qPCR (94.2%), and between IHC-1 and qPCR (83.9%). Correlations between IHC-1 and SEQ and between IHC-2 and SEQ were 71.5% and 76.2%, respectively. The IHC-2 protocol had higher sensitivity, PPV, and NPV, and Cohen’s kappa than IHC- 1. The presence of *BRAF* V600E mutation in IHC-2 statistically correlated with age at diagnosis, histopathological stage, and extrathyroidal extension.

**Conclusions:**

The results obtained in this study indicate a lack of concordance between *BRAF* V600E detection by IHC and molecular methods. The IHC method cannot replace molecular methods for the detection of the *BRAF* V600E mutation.

## INTRODUCTION

Papillary thyroid carcinomas are the most common thyroid cancers. The most common variants include classical, follicular variant, and tall cell variant. Less common variants include oncocytic, columnar cell, diffuse sclerosing, and solid forms [1]. Numerous studies of the molecular mechanisms underlying PTC have enabled identification of oncogenic driver mutations in 96.5% cases [2]. One such driver mutation, *BRAF* V600E, is detected at various frequencies, depending on the commercially available method applied and other factors, such as the demographic and disease characteristics of patients. Hence, its reported frequency of occurrence oscillates between as low as 28.2% [3] and up to 90% (reported by Korean studies) [4, 5].

The role of the *BRAF* V600E mutation as a prognostic factor is not clearly defined. Numerous studies have reported correlations between *BRAF* V600E mutation and unfavorable clinical and pathological features, including association with reduced survival rates [4, 6-11]; however, some researchers have challenged the role of *BRAF* V600E mutation as an indicator of poor prognosis [12-16]. Recent American Thyroid Association recommendations provide for *BRAF* status (if known), together with other prognostic factors, in risk stratification of PTC clinical course [17].

DNA-based analyses are used as standard for detection of the *BRAF* V600E mutation in thyroid carcinoma [18]. Diverse molecular methodology is employed for *BRAF* V600E detection in routine clinical practice, including pyrosequencing, real-time PCR (qPCR), allele-specific PCR (ASA-PCR), and Sanger sequencing (SEQ) [5, 12, 19-20]. Recently, a method to detect the *BRAF* V600E mutation by immunohistochemistry (IHC), using the mouse monoclonal antibody, clone VE1, was developed [21-27]. Despite the high sensitivity and specificity of IHC, it remains unclear whether it can replace molecular testing in clinical practice.

The aim of this paper was to compare the frequency of detection of *BRAF* V600E mutations in patients with PTC by two alternative staining IHC protocols using the VE1 monoclonal antibody, and the molecular methods, SEQ and qPCR. We evaluated the concordance of the results obtained using the various methods, and assessed the correlation of both positivity for the *BRAF* mutation by IHC, and IHC staining intensity, with clinical and pathological features of PTC.

## RESULTS

### Comparison of results obtained using the two IHC protocols (IHC-1 and IHC-2)

Frequency of mutation detection, staining intensity, and percentage of cells staining positive for the *BRAF* V600E mutation were compared between the two IHC methods, IHC-1 and IHC-2.

#### Frequency of mutation detection and analysis of concordance

The *BRAF* V600E mutation was detected in 57.1% of patients (80/140) using the IHC-1 protocol, whereas 62.9% of patient samples (88/140) were positive using IHC-2. The difference in the frequency of mutation detection between the two methods bordered on statistical significance (P = 0.06). The concordance in *BRAF* V600E mutation detection using protocols IHC-1 and IHC-2 was 90% (126/140). In 14 cases, the results obtained using the two methods were inconsistent; for 11 cases, the *BRAF* V600E mutation was detected only using IHC-2, while for three cases the mutation was only detected by IHC-1. Cohen’s kappa was 0.79 (95% confidence interval, 0.63–0.96), indicating substantial agreement between IHC-1 and IHC-2.

#### Percentage of stained cells and analysis of concordance

The IHC protocols differed from one another with regard to the percentage of stained cells observed. The longer incubation time in the IHC-2 protocol correlated with a larger number of cases where the percentage of stained cells was considerably higher than that observed using IHC-1. The percentage of cells stained was more frequently recorded as 100% for samples evaluated using IHC-2 than for those tested with IHC-1, with 72 of 140 preparations (51.4%) vs. 45 of 140 preparations (32.1%), respectively (P < 0.0001). Concordance between the two protocols in the percentage of stained cells was noted in 90 of 140 preparations (64.3%).

#### Staining intensity and analysis of concordance

The longer sample incubation time in IHC-2 also led to increased numbers of cases with stronger staining intensity scores using this protocol. Staining intensity scores of +3 were recorded more frequently for samples evaluated using IHC-2 than for those evaluated using IHC-1, with 53 of 140 samples (37.9%) vs. 31 of 140 samples (22.1%) (P = 0.0002). Concordance in staining intensity scores between the two methods was noted for 90 of 140 samples (64.3%). Concordance of the two methods regarding both the percentage of cells staining positive and staining intensity was observed for only 73 samples (52.1%).

### Comparison of BRAF V600E mutation detection using IHC and molecular methods, including analysis of concordance

*BRAF* V600E mutations were detected most frequently using the IHC-2 (n = 88) and qPCR (n = 82) methods, and least often by SEQ (n = 53). Poor DNA quality led to a lack of results generated by SEQ and qPCR methods for 10 (7.1%) and 3 (2.1%) cases, respectively. These data indicate that the qPCR method is less sensitive to DNA quality than SEQ, although the difference was not statistically significant (P = 0.096) (Tables 1 and 2). Concordance in detecting BRAF V600E mutation between these two molecular methods SEQ vs qPCR was noted for 102 of 140 samples (72,9%). However, qPCR method is more sensitive, as it can detect mutations present at frequencies as low as 1% alleles [4]. qPCR detected the BRAF V600E mutation in 51 cases, where the mutation was detected by SEQ, in 24 where SEQ did not reveal the mutation and in 7 cases which were not tested for the BRAF V600E mutation by SEQ due to degeneration of samples.

**Table 1:** Comparison of IHC-1 with molecular biology methods for detection of the BRAF V600E mutation in patients with PTC (n = 140)

	Method			P value*		
	IHC-1	SEQ	qPCR	IHC-1 vs. SEQ	IHC-1 vs. qPCR	SEQ vs. qPCR
p.V600E mutation	80 57.1%	53 37.9%	82 58.6%	<0.0001	0.84	<0.0001
WT	60 42.9%	77 55.0%	55 39.3%	0.001	0.40	0.0001
DNA degradation	NA NA	10 7.1%	3 2.1%	NA	NA	0.096

Notes: *McNemar test.

Abbreviations: NA, not applicable (due to DNA degradation; does not refer to the IHC method); IHC-1, immunohistochemistry protocol 1 (incubation of samples with VE1 primary antibody, for 16 min); SEQ, Sanger sequencing; qPCR; real-time PCR; WT, wild-type.

**Table 2:** Comparison of IHC-2 with molecular biology methods for detection of the BRAF V600E mutation in patients with PTC (n = 140)

	Method			P value*		
	IHC-2	SEQ	qPCR	IHC-2 vs. SEQ	IHC-2 vs. qPCR	SEQ vs. qPCR
p.V600E mutation	88	53	82	<0.0001	0.11	<0.0001
	62.9%	37.9%	58.6%			
WT	52	77	55	<0.0001	0.44	0.0001
	37.1%	55.0%	39.3%			
DNA degradation	NA	10	3	NA	NA	0.096
	NA	7.1%	2.1%			

Notes: *McNemar test.

Abbreviations: NA, not applicable (due to DNA degradation; does not refer to the IHC method); IHC-2, immunohistochemistry protocol 2 (incubation of samples with VE1 primary antibody, for 32 min); SEQ, Sanger sequencing; qPCR; real-time PCR; WT, wild-type.

Comparison of the results obtained by IHC-1 and SEQ, and IHC-1 and qPCR, indicated concordance rates of 71.5% and 83.9%, respectively, while concordance rates between results generated by IHC-2 and SEQ, and IHC-2 and qPCR, were 76.2% and 94.2%, respectively. Hence, the highest concordance was between IHC-2 and qPCR, where differences were observed in only eight cases; six of these eight discordant samples were positive by IHC-2 and negative by qPCR, and two were positive by qPCR and negative by IHC-2. For an additional three cases positive by IHC-2, no result was obtained using qPCR, due to degradation of the DNA sample; these three cases were not included in the analysis. The remaining eight discordant cases were subjected to additional evaluation by NGS. The results of NGS were fully concordant with those of qPCR (in two cases, mutations were confirmed, while in six negative results for *BRAF* V600E mutation were obtained). Based on these results, the qPCR method was selected as the gold standard for detection of *BRAF* V600E mutations.

### Analysis of the quality of IHC testing for detection of the BRAF V600E mutation compared with qPCR

Based on the results presented above, qPCR was assumed as the gold standard method, and the following characteristics of both IHC assays were calculated in relation to it: sensitivity, specificity, accuracy, positive predictive value, and negative predictive value. For all characteristics, except for specificity, IHC-2 was superior to IHC-1 (Table 3).

**Table 3:** Performance evaluation of IHC-1 and IHC-2 methods in comparison with qPCR

	IHC-2 vs. qPCR	IHC-1 vs. qPCR	P value
Sensitivity	97.6% (95% CI: 91.5–99.7%)	84.1% (95% CI: 74.4–91.3%)	0.001^a^
Specificity	89.1% (95% CI: 77.8–95.9%)	83.6% (95% CI: 71.2–92.2%)	0.083^a^
Positive predictive value	93.0% (95% CI: 85.4–97.3%)	88.5% (95% CI: 79.2–94.6%)	0.020^b^
Negative predictive value	96.1% (95% CI: 86.5–99.5%)	78.0% (95% CI: 65.3–87.7%)	0.001^b^
Accuracy	94.2% (95% CI: 88.8–97.4%)	83.9% (95% CI: 76.7–89.7%)	-
Cohen’s kappa	0.88 (95% CI: 0.71–1.00)	0.67 (95% CI: 0.50–0.84)	-

Notes: ^**a**^McNemar’s test; ^**b**^Weighted generalized score statistic method.

Abbreviations: IHC-1, immunohistochemistry protocol 1 (incubation of samples with VE1 primary antibody, for 16 min); IHC-2, immunohistochemistry protocol 2 (incubation of samples with VE1 primary antibody, for 32 min); qPCR; real-time PCR; CI, confidence interval.

### Evaluation of the correlation between clinical and pathological features and BRAF V600E mutation detected using IHC-2

Associations between the *BRAF* V600E mutation detected by the IHC-2 method and the clinical and pathological features of PTC are presented in Table 4. Univariate analysis suggested statistically significant correlations between mutation detection by IHC-2 and age at diagnosis, tumor stage (pTNM), and extrathyroidal extension. Mutations were observed more frequently in older patients (≥45 years). The average age of patients whose tumors harbored mutations was 55 years, while that of patients in whom the mutation was not detected was 47 years (P = 0.0002). Higher histopathological tumor grade (P = 0.01) and extrathyroidal extension (P = 0.047) were also more frequent in patients with the mutation. No statistically significant correlation was observed with any other features analyzed. By contrast, by multivariate analysis only the association with age at diagnosis was statistically significant (Table 4). The results obtained were concordant with those generated by analysis of associations with *BRAF* V600E mutation determined by qPCR (data not shown).

**Table 4:** Univariate and multivariate analyses of the correlation between the clinical and pathological features of papillary thyroid carcinoma and mutation status evaluated using **IHC-2**

	IHC-2 (+)	IHC-2 (-)	Univariate analysis			Multivariate analysis		
	N = 88	N = 52	P value	OR	95% CI	P value	OR	95% CI
Age (years)								
<44	13 (14.8%)	23 (44.2%)		1			1	
≥45	75 (85.2%)	29 (55.8%)	0.0002	4.6	2.0–10.2	0.001	4.4	1.8–10.6
Sex								
Female	76 (86.4%)	46 (88.5%)		1				
Male	12 (13.6%)	6 (11.5%)	0.72	1.2	0.4–3.4			
Tumor size								
≤10 mm	40 (45.5%)	20 (38.5%)	0.42	1.3	0.7–2.7			
>10 mm	48 (54.5%)	32 (61.5%)		1				
Vascular invasion								
No	84 (95.5%)	47 (90.4%)	0.25	2.2	0.6–8.7			
Yes	4 (4.5%)	5 (9.6%)		1				
Multifocality								
No	64 (72.7%)	44 (84.6%)		1				
Yes	24 (27.3%)	8 (15.4%)	0.11	2.1	0.8–5.0			
Extrathyroidal extension								
No	55 (62.5%)	41 (78.8%)		1			1	
Yes	33 (37.5%)	11 (21.2%)	0.047	2.2	1.01–4.9	0.17	2.1	0.7–6.1
pTNM								
I–II	51 (58.0%)	41 (78.8%)		1			1	
III–IV	37 (42.0%)	11 (21.2%)	0.01	2.7	1.2–6.0	0.67	1.3	0.4–3.8
ATA risk								
Low	51 (58.0%)	36 (69.2%)		1				
Moderate or high	37 (42.0%)	16 (30.8%)	0.19	1.6	0.8–3.4			
Final status								
No remission	5 (5.7%)	4 (7.7%)		1				
Remission	83 (94.3%)	48 (92.3%)	0.64	1.4	0.4–5.4			

Abbreviations: IHC-2, immunohistochemistry protocol 2 (incubation of samples with VE1 primary antibody, for 32 min); OR, odds ratio; CI, confidence interval; pTNM, tumor grade based on size and extent of main tumor, local lymph node invasion, and metastasis; ATA, American Thyroid Association Guidelines.

### Evaluation of the correlation between the clinical and pathological features of PTC and BRAF V600E staining intensity determined by IHC-2

The staining intensity determined using IHC may indicate the amount of mutated protein present in tumors and could, therefore, facilitate quantitative analysis of the effect of the *BRAF* V600E mutation on the clinical features of PTC. An evaluation was performed to assess correlations between staining intensity and clinical features. Higher staining intensity was not statistically significantly correlated with any clinical or pathological disease features; thus our data do not confirm any additional benefits of detection of the *BRAF* V600E mutation using IHC methods.

## DISCUSSION

Until recently, *BRAF* V600E mutation status was solely evaluated using molecular biology methods. [12, 27]. With the development of the V600E antibody, which detects only the mutated protein, an opportunity arose to evaluate mutation status using routine histopathological techniques [28, 29]. At present, an intense discussion is ongoing in the literature regarding the usefulness of IHC for analysis of *BRAF* V600E and the concordance of the results it generates with those obtained using molecular tests. In this paper, the frequency of *BRAF* V600E mutation detection using two IHC protocols was evaluated, and an analysis of the concordance of the resulting diagnoses with those generated using molecular methods (SEQ, qPCR) was performed.

Using IHC-1, mutations were detected in 80 cases (57.1%), whereas use of the alternative protocol, IHC-2, increased the number of identified mutations to 88 (62.9%). These results indicate a significant effect of IHC test methodology on the effectiveness of the technique for mutation detection. Some researchers fail to describe the details of the methodology used in their studies, making it difficult to compare results generated using different protocols [16, 22, 30].

The frequency of BRAF V600E mutation detection with the use of IHC in PTC is assessed at 52-88,5% [16, 22, 23, 25, 30, 31, 32, 33]. The research that was mentioned uses different criteria for determining whether the result is positive or negative (the differences concern both the staining intensity, as well as the number of stained cells). A different approach towards the cut-off value for the staining reaction that determines a positive outcome makes the comparison of the results difficult.

In the aforementioned studies, patients with various PTC variant tumors were tested, different criteria were applied to define positive results, and two different anti-*BRAF* V600E antibodies were used: the VE1 anti-*BRAF* V600E antibody (Spring Bioscience), and in studies [23, 30, 32, 33] the VE1 anti-*BRAF* V600E hybridoma antibody [25]. Various protocols, different to the one employed by the present investigation, may also have been used, although a number of studies [23, 25, 30, 32] also reported use of the Ventana Medical System, the latest version of which (BenchMark Ultra) was used for the present work. Only Dvorak et al. [24] both used the same type of antibody and applied the Ventana BenchMark XT (Ventana Medical Systems Inc.) staining device in their study, comparable with the present investigation. These investigators reported that the *BRAF* V600E mutation was detected in 71.23% (52/73) of PTC cases, which may be a consequence of differences in the characteristics of the groups under study, since Dvorak et al. included samples from various PTC histological subtypes, in addition to differences in the study protocols, as they used an older version of the Ventana BenchMark apparatus.

Differences in the frequency of detected mutations may also result from the amount of VE1 antibodies applied to stain samples. Ilie et al. diluted the VE1 antibody 1:10 [25], while we used it at a concentration of 1:100, and Ghossein et al. [30] and Martinuzzi et al. [32] report working dilutions of 1:50. The frequencies of mutation detection using the 1:50 dilutions were 68.75% in the classical form of PTC and 70.9% in all PTC, compared with 62.9% of patients with classical PTC in the present study using a dilution of 1:100. These results may indicate a linear relationship between the concentration of VE1 antibody and the frequency of mutation detection by IHC. It is important to note that, in the present work, neither the study protocol nor the anti-*BRAF* V600E antibody (developed by Roche Diagnostics) was modified in any way.

In this study, the concordance between diagnoses obtained by IHC and molecular methods depended on both the IHC protocol used and the sensitivity of the molecular method. The IHC-1 protocol demonstrated 71.5% concordance with SEQ, and 83.9% with qPCR, while IHC-2 showed 76.2% and 94.2% concordance, respectively, using the same molecular methods. By contrast, Koperek et al. (SEQ) [16], Routhier et al. (SNAaPshot) [22], Capper et al. (SEQ) [29], and Ghossein et al. (MassArray) [30] reported 100% concordance with the molecular methods applied in their studies.

Zagzag et al. [23] reported that the SEQ method was more sensitive than IHC, detecting 76% (28/37) cases of *BRAF* V600E mutation, with a concordance of 89.3%. In the present study, the SEQ method detected the mutation in 37.9% of patients and was less sensitive than IHC (Tables 1 and 2). Results similar to ours were obtained by Zhu et al. [31], who found that the IHC method was more sensitive than SEQ/Amplification refractory mutation system (ARMS), with the *BRAF* V600E mutation detected in 68.6% (81/118) vs. 61.9% (73/118) of PTC cases, respectively; the overall concordance between IHC and SEQ/ARMS was 93.2%. Ilie et al. [25] detected the *BRAF* V600E mutation in 79% of cases, using at least one of three molecular methods (SEQ, pyrosequencing, and SNaPshot), and higher IHC concordance was observed between pyrosequencing and SNAaPshot, while IHC was more sensitive than SEQ, detecting the mutation in 78% (151/194) vs. 73% (142/194) of cases, respectively. Martinuzii et al. [32] determined 80% concordance between IHC and two molecular methods (SEQ and PNA-clamp qPCR), whereas comparison of the two methods showed higher concordance between IHC and the molecular method with higher sensitivity PNA-clamp qPCR (92%) than between PNA-clamp qPCR and SEQ (86%), similar to the present study.

In this study, we demonstrated that identification of the *BRAF* V600E mutation by IHC correlated with patient age at diagnosis, extrathyroidal extension, and lower histopathological grade (pTNM), whereas Zagzag et al. only identified an association with extrathyroidal extension [23]. Similar to our results, Na Ji et al. [33] demonstrated statistically significant correlations between *BRAF* V600E mutation ascertained by IHC and both pTNM stage and extrathyroidal extension. The cited study also evaluated the correlation of the mutation with multifocality, metastases to lymph nodes, distant metastases, histological PTC type, degree of desmoplasia, and lymphocytic infiltration of the tumor [33].

In contrast to our findings, Koperek et al. found no correlation between *BRAF* V600E mutation detected by IHC and extrathyroidal extension [16], whereas they also observed a statistically significant increased frequency of the *BRAF* V600E mutation in older patients, consistent with our results. Abd Elmageed et al. [34] found no correlation between *BRAF* V600E mutation and patient age or extrathyroidal extension, although they report a strong correlation between the mutation and histological PTC type and original tumor size. In cited investigations, various PTC subtypes were analyzed; hence the groups under study differed somewhat from our patient cohort, which was composed only of individuals with classical PTC.

There are few investigations where the accuracy of mutation detection by IHC and molecular methods was verified using NGS [24, 29, 32]. Our results indicate full concordance between qPCR and NGS data. False-negative and false-positive results may occur while detecting the BRAF V600E mutation with IHC. False-positive results may have occurred due to insufficient specificity of the antibody, or suboptimal conditions used to fix histopathological preparations. All eight cases in our study in which the discrepancy between IHC-2 and qPCR was detected, as well as the rest of the study group, were from the same period time and did not differ in histopathology.

Six of our cases were positive by IHC-2 but negative by qPCR. Such IHC false positives are more difficult to explain. It is unlikely that qPCR lack enough sensitivity to detect low level of mutated gene in this context especially that qPCR test is less vulnerable to DNA degradation due to very short fragment of DNA amplified (67bp) [35]. Described several reasons for false positive results of IHC staining: nonspecific background signal, endogenous peroxidase, the use of inappropriately high antibody concentrations, pigment mistaken for true signal, endogenous biotin, drying artifact, and ‘‘pseudospecific’’ signal [36]. Lately proved that false positive results could be related to horseradish peroxidase conjugates [37].

False-negative findings may be explained by ischemia in tumor tissues, which can lead to disruption of transcription or translation and, consequently, to the lack of generation of mutated protein, despite the presence of the mutation, or the occurrence of additional mutations hindering mRNA translation into active protein [24, 25]. Two cases in our study with IHC-2 negativity and qPCR positivity represents true false negative which probably arise from the loss of expression of the mutant antigen. In our cases the reason for the lack of staining could be due to suboptimal fixation conditions. There was not necrotic areas which in the manifestation of tissue ischemia, what has been shown to reduce BRAF V600E protein expression and is a potential source of false negative. It is also possible that additional mutations may prevent translation of the mRNA into the functional protein. In this two cases there were no positive cell population.

Another potential explanation for the lack of concordance could be insufficient numbers of cancerous cells in the samples analyzed, leading to the predominance of the wild-type allele over the mutated form, precluding mutation detection, even using the most sensitive molecular methods. However in this study all samples have more than 10% of tumor cells in the area used for DNA isolation.

In conclusion, the results obtained in this study indicate a lack of concordance between *BRAF* V600E detection by IHC and molecular methods. Consequently, we conclude that IHC cannot replace molecular analyses for identification of this mutation. Since we obtained both false-positive and false-negative results using IHC, this method is also unsuitable for preliminary screening for positive samples for subsequent verification by molecular methods. Moreover, we observed no additional benefits of detection of *BRAF* V600E by IHC, since there were no significant correlations between staining intensity and the clinical and pathological disease features.

## MATERIALS AND METHODS

### Study design and patients

This was a pilot study including 140 patients. As one of the study objectives was to evaluate the relationship between clinical course and *BRAF* mutation status determined by IHC, all patients with at least 10 years of follow-up with pT3, pT4 and lower clinical stages were included in the study. Patients were treated in the same center, (in the Clinic of Endocrinology, Holycross Cancer Centre, Kielce), diagnosed with classical PTC between 2000 and 2005, and recruited to the trial during routine follow-up from 2012 to 2014. Patients provided their written consent for molecular tests to be performed. Archival paraffin blocks of thyroid cancer constituted the material for analyses. All cases were obtained from our Pathology Department at Holycross Cancer Center and were always prepared in the same way. After dissection, all gross specimens were placed in 10% phosphate-buffered formalin for 24 hours in room temperature before undergoing automated processing. At the end of the processing step, the tissue-containing cassettes were immersed in paraffin. The “blocks” were mounted on a microtome and 4-μm sections were cut from the surface of each block. For HE staining, plain glass slides were used, but for immunohistochemistry, special glass slides with an adhesive surface (FLEX IHC microscope slides DAKO) were used.

The average observation time after diagnosis was 12 years (standard deviation, 18 months; median, 12 years). The clinical characteristics of the patients are presented in Table 5. The trial was approved by the Bioethics Committee at the Regional Chamber of Physicians.

**Table 5:** Clinical and pathological characteristics of patients with PTC (n = 140)

Feature*	Total (n = 140)
Age at diagnosis (years)	51.8 (12.3) [15–76; 52]
Diameter of dominant tumor (mm)	17.3 (15.0) [0.7–80; 12]
Sex	122 (87.1)
Female	
Male	18 (12.9)
Vascular invasion	131 (93.6)
No	
Yes	9 (6.4)
pT	72 (51.4)
T1	
	17 (12.1)
T2	
	51 (36.4)
T3–T4	
N	117 (83.6)
N0	
N1	23 (16.4)
M	135 (96.4)
M0	
M1	5 (3.6)
pTNM	84 (60.0)
I	
	8 (5.7)
II	
	48 (34.3)
III–IV	
Multifocality	108 (77.1)
No	
Yes	32 (22.9)
Extrathyroidal extension	96 (68.6)
No	44 (31.4)
Yes	

Notes: *Age at diagnosis and diameter of dominant tumor are presented as mean (standard deviation) [min–max; median], and other features are presented as n (%).

Abbreviations: pT, size and extent of main tumor; N, local lymph node; M, metastasis; pTNM, tumor grade based on pT, N, and M.

#### Immunohistochemistry

**Figure 1:**
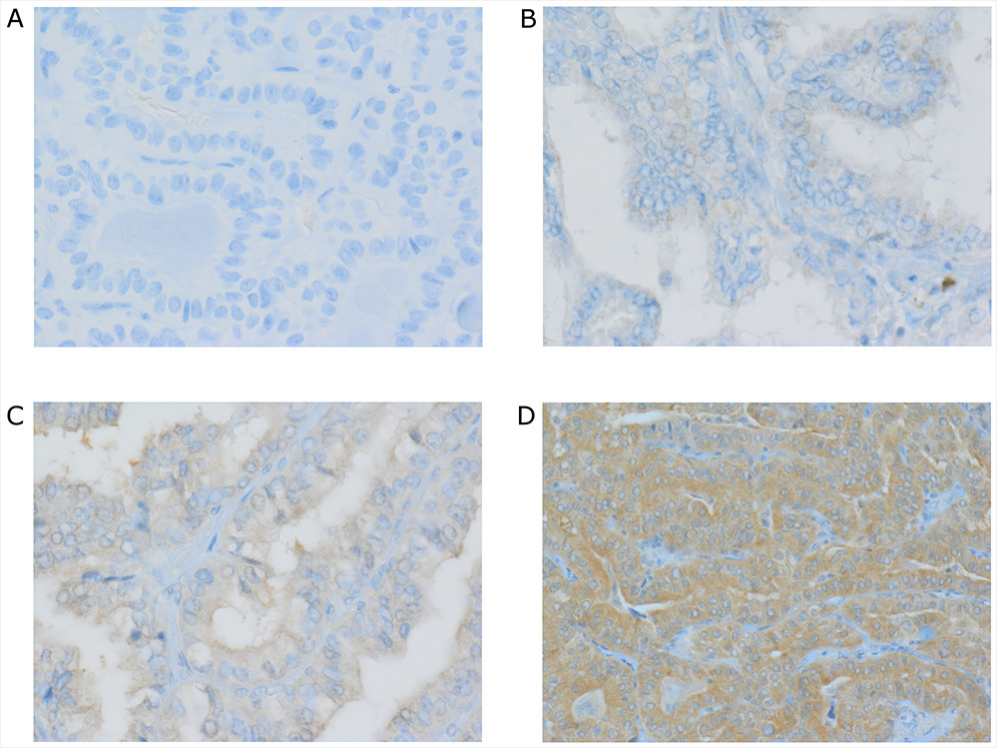
BRAF V600E immunohistochemistry using VE1 antibody **(A)** Negative classical PTC. **(B – D)** Positive classical PTC scored respectively as weak +1 (B), moderate +2 (C), strong +3 (D). Representative IHC staining of positive and negative expression of BRAF is presented at 100x magnification. Magnification images were taken on Olympus AX60 microscope with CS-D (Olympus Soft Imagining Solutions GMBH, Germany).

Freshly cut sections (4 μm thick) of formalin-fixed, paraffin-embedded tissue samples from 140 patients with PTC were incubated in an oven at 62°C for 20 min. Subsequently, they were stained with mouse monoclonal *BRAF* V600E antibody (1/100 titer; clone VE1, Ventana). Briefly, staining was performed on the Ventana BenchMark Ultra (Ventana Medical Systems Inc.) using one of two different protocols (IHC-1 and IHC-2) according to producent’s recommendation. They differ in incubation time. The staining protocol included online deparaffinization, HIER (Heat Induced Epitope Retrieval) with Ventana Cell Conditioning 1 for 32 min, and primary antibody incubation for 16 min at 37°C (IHC-1), and 32 min (IHC-2). A sample from a skin malignant melanoma known to carry the *BRAF* V600E mutation was used as a positive control. As a negative control, the primary antibody was replaced with non-immune animal serum, diluted to the same concentration as the primary antibody. Antigen-antibody reactions were visualized using a Ventana OptiView™ Amplification kit, followed by a Ventana OptiView™ Universal DAB Detection Kit (Optiview HQ Linker 8 min, Optiview HRP Multimer 8 min, Optiview Amplifier H_2_O_2_/Amplifier 4 min, Optiview Amplifier Multimer 4 min, Optiview H_2_O_2_/DAB 8 min, Optiview Copper 4 min). Counterstaining was performed using Ventana Hematoxylin II for 8 min, followed by bluing reagent for 4 min. Finally, all slides were removed from the stainer and dehydrated, and coverslips were applied, for microscopic examination. IHC slides were first scored by the primary pathologist and subsequently by two additional independent pathologists. All three pathologists were blind to the molecular results. The primary pathologist (JK) was most intimately involved with the development of the immunostaining method and the most experienced and familiar with variations in staining intensity and distribution, not only among PTC cases, but also in positive and negative controls. Given the level of expertise of the primary pathologist in interpretation of *BRAF* V600E immunostaining, and the sequence of events, the interpretation of the primary pathologist was considered the “gold standard” with which the results of the *BRAF* V600E molecular mutation analyses were compared (see below). The results obtained by three pathologists in scoring of *BRAF* V600E IHC were compared. Cases were scored as positive (“IHC-positive”) or negative (“IHC-negative”); only tumor cells showing non-ambiguous cytoplasmic staining for *BRAF* V600E were scored as positive. The intensity of VE1 immunostaining was scored on a scale from 0 to 3, with 0 as negative, 1 as weak, 2 as moderate, and 3 as strong cytoplasmic staining. Representative scores of all immunostainings are shown in Figure 1. The proportion of stained tumor cells (0–100%) was also recorded. Cases were classified as positive when signal intensity was ≥1 and the percentage of labeled cells was ≥25%, except for one case, where signal intensity was moderate [2] but the proportion of the sample stained was 10% using protocol IHC-2. In this particular case, the majority of the tumor area was infarcted, probably after an earlier fine needle aspiration biopsy procedure. In case of discordant results between IHC-1 and IHC-2 we reiterated IHC staining repeatedly and our results were the same.

### Molecular methods

Molecular genetic studies (SEQ, qPCR, and Next Generation Sequencing) were performed as previously described [12, 38, 39].

#### DNA isolation

The pathologist marked the area containing PTC tumor cells on a hematoxylin- and eosin-stained slide. In all studied cases in the marked area tumor cell content was more than 10%. Then, the tumor tissue on matched unstained slides was deparaffinized and the pathologist-selected area was transferred to a tube for DNA isolation using the Maxwell 16 and Maxwell® 16 FFPE Tissue LEV DNA Purification Kit, according to the manufacturer’s instructions (Promega, USA). The isolated DNA concentration was measured by using NanoDrop (TkBiotech, Warsaw. Poland). Mean concentration of isolated DNA was 100ng/ul and mean purity: 260/280 1,8-2,0.

#### Sanger sequencing (Seq)

We amplified a 224 bp segment of BRAF exon 15 containing codon 600 using the following PCR primers: BRAFek15f (5’-TCATAATGCTTGCTCTGATAGGA-3’) and BRAFek15r (5’-GGCCAAAAATTTAATCAGTGGA-3’). After purification of the PCR products, sequencing was performed using a BigDye Terminator v1.1 Cycle Sequencing kit (Life Technologies, Warsaw, Poland) and an ABI 3130 Automatic Capillary DNA Sequencer (Applied Biosystems, USA).

#### Real-time PCR (qPCR)

qPCR assay targeting a 68 bp region of BRAF exon 15 performed using Rotor-Gene Q (Qiagen, Syngen-Biotech, Poland) with the primers, forward 5’-AGACCTCACAGTAAAAATAGGTGATTTTGG-3’ and reverse 5’-GATGGGACCCACTCCATCG-3’, and BRAF mutant-specific (6FAM-CTACAGAGAAATC-MG-BNFQ) and BRAF WT allele-specific (VIC-CTACAGTGAAATC-MGB-NFQ) probes. Our qPCR test was used in this study as a qualitative analysis.

#### Next generation sequencing

#### Library preparation

10 ng of DNA (Qubit 2.0 device -Thermo Scientific, USA) from each sample was added to the multiplex PCR reaction for library preparation using the Ion AmpliSeq Library Kit 2.0, Ion AmpliSeq Cancer Hotspot Panel v2 Kit (CHPv2), according to the manufacturer’s instructions (Thermo Fisher Scientific). CHPv2 contains 207 pairs of primers, covering hotspots in the 50 most often mutated genes in cancer. The products resulting from the multiplex PCR were subjected to partial enzymatic digestion to remove the primers and the adapters with barcodes were enzymatically attached (Ion AmpliSeq Library Kit 2.0, Thermo Fisher Scientific) to the multiplex PCR products using the Ion Xpress Barcode Adapters 1-32 Kit (Thermo Fisher Scientific). The prepared libraries were purified by two rounds of Agencourt AMPure XP (Beckman Coulter Genomics) according to the instructions of the manufacturer of the Ion AmpliSeq Library Kit (Thermo Fisher Scientific).

### Preparation of clonally amplified template for sequencing - emulsion PCR (emPCR)

The concentration of each 8 barcoded library was measured by real-time PCR using the Library Ion Quantitation Kit (Thermo Fisher Scientific). Then barcoded libraries were mixed in equimolar proportions (20 pM dilutions, eight libraries per pool) and subjected to emulsion PCR (emPCR). The emPCR reaction and the following enrichment step were performed according to the manufacturer’s instructions using the Ion OneTouch 2 System and Ion PGM Template OT2 200 kit (Thermo Fisher Scientific).

#### Sequencing

The obtained barcoded clonally amplified libraries were loaded onto 318 chip and sequenced using the Ion PGM Sequencing 200 Kit v2 and IonTorrent Personal Genome Machine according to the manufacturer’s instructions (Thermo Fisher Scientific).

#### NGS data analysis

The raw data generated during sequencing was processed using the Torrent Server Suite 4.2 (Thermo Fisher Scientific). The obtained sequences were aligned (mapped) to the reference sequence of the human genome (hg19) with the Torrent Server Suite 4.2. Variant calling was performed by Variant Caller v4.2 embedded in the Torrent Server Suite 4.2. Default parameters used for CHPv2 data analysis were: minimum allele frequency - SNP = 0.02 / INDEL = 0.05, minimum quality -10 and minimum coverage - 20.

Analysis of next generation sequencing (NGS) data focused on amplicons covering the *BRAF* V600 codon, which were reviewed using Integrative Genomics Viewer [40]. The mean coverage for *BRAF* amplicon was 550x (range:150x-800x).

### Statistical analyses

Quantitative data are presented as means and standard deviations, medians, and ranges, whereas categorical data are presented as numbers and percentages. IHC and molecular methods for the detection of the *BRAF* mutation were compared by calculating sensitivity, specificity, positive predictive value, negative predictive value, overall percent agreement (the number of concordant cases divided by the total number of evaluated cases), and Cohen’s kappa measure of agreement. Paired proportions were compared using McNemar’s test. Tests for differences in positive and negative predictive values of two diagnostic methods were performed using the weighted generalized score statistic proposed by Kosinski [41]. Univariate and multivariate logistic regression models were used to evaluate the association of clinical factors with *BRAF* mutation. Two-tailed P values <0.05 were considered statistically significant. Computations were performed using the statistical packages, R (version 3.3.2) [42], epiR [43], DTComPair [44], and STATISTICA (version 12).

## Abbreviations

PTC: papillary thyroid carcinoma
IHC: immunohistochemistry
SEQ: sanger sequencing
qPCR: quantitative polymerase chain reaction
NGS: next generation sequencing
